# Predictors of Romanian Psychology Students’ Intention to Successfully Complete Their Courses—A Process-Based Psychology Theory Approach

**DOI:** 10.3390/bs13070549

**Published:** 2023-07-01

**Authors:** Ioana-Eva Cădariu, Dana Rad

**Affiliations:** 1 Department of Psychology, West University of Timisoara, 300223 Timisoara, Romania; 2 Department of Psychology, Tibiscus University of Timisoara, 300223 Timisoara, Romania; 3 Institute of Psychotherapy Psychological Counselling and Clinical Supervision, 300223 Timisoara, Romania; 4 Center of Research Development and Innovation in Psychology, Faculty of Educational Sciences Psychology and Social Sciences, Aurel Vlaicu University of Arad, 310032 Arad, Romania

**Keywords:** academic adjustment, academic self-efficacy, satisfaction with university, first-year students, mediation

## Abstract

Student retention is a frequently researched issue due to the incidence of student dropout and its significance to learning outcomes. However, there are research gaps that need to be addressed in understanding the factors influencing student dropout in the context of higher education in Romania. This cross-sectional investigation aims to fill these gaps by examining the relationships between satisfaction with the specialization, self-regulation of learning behavior, students’ perceived stress, perceived acceptance from family and friends, and the intention to complete studies. The study utilizes various statistical analysis techniques, including mediation analysis and correlation analysis, to analyze the collected data. An online questionnaire was administered to non-randomized students majoring in Psychology, and a total of 144 valid and consented responses were obtained. The results reveal significant influences of satisfaction with the specialization, self-regulated learning, and students’ perceived stress on the intention to successfully complete courses. Furthermore, academic self-efficacy was found to fully mediate the relationship between satisfaction with the specialization and academic adjustment. These findings contribute to a better understanding of the student dropout process in the Romanian higher education system. By identifying the factors associated with student retention, this study provides insights that can inform the development of interventions aimed at improving students’ retention and overall learning outcomes.

## 1. Introduction

Student retention is a widely researched phenomenon with significant implications for learning outcomes in the field of education [1,2,3,4]. However, theoretical progress in understanding student retention has been limited, with many studies lacking a solid theoretical framework [4]. Existing research often focuses on factors related to academic and social integration, without considering the dynamic nature of student persistence [5]. Moreover, traditional sociological models of student persistence have shown limited success in predicting voluntary student dropout [4]. To address these gaps, there is a need to explore new process-based psychology theories that can better explain the conscious decision-making process behind student dropout [4].

Early identification of students at risk of dropout has been recognized as crucial for developing effective interventions to improve retention rates [6,7,8]. Previous research has demonstrated that factors associated with students’ academic and social integration have limited predictive power in determining students’ intentions to leave educational settings [4]. However, the theory of planned behavior has shown promise in explaining a significant proportion of the variation in students’ desire to quit [4].

There is a need for research to better understand student retention behavior within this setting. This study aims to investigate the intentions of Romanian students to continue their Psychology courses and program using a process-based psychology theory. The findings of this study can inform policymakers in developing programs and initiatives to enhance student persistence rates and improve learning outcomes [9,10]. Specifically, the study aims to shed light on the phenomenon of student dropout in the context of Romanian higher education, contributing to a better understanding of the factors influencing student retention.

By adopting a process-based psychology approach and exploring the specific determinants of student persistence, this study aims to fill the existing gaps in the literature and provide valuable insights into the complex nature of student dropout. The implications of this research extend beyond academia, as the findings can inform interventions and strategies to support students’ educational journeys and enhance their overall success.

### Literature Review

The transition from high school to college leads to a number of significant changes in young people’s lives; as new responsibilities arise, students must allocate time to accomplish academic tasks, and must make efforts to develop new relationships with college peers [11]. Students who adapt to the university environment will have higher grades and are less likely to suffer from depression [12,13]. Insufficient clarity regarding the nature and expectations of college education is a prevalent issue among many students, leading to unmet expectations and subsequently influencing their decision to discontinue their studies [14].

The specialized literature has introduced the concept of college adaptation [15]. Four categories—academic adjustment, social adjustment, personal–emotional adjustment, and institutional attachment—are used by the two researchers to categorize college adjustment. The extent to which students have adjusted to academic obligations, their devotion to the material being taught, and their attempts to finish assignments are all reflected in their academic adjustment. Social adaptation reflects how well a student has adapted to the academic environment, participates in campus activities, has the capacity to make new acquaintances, and does not experience social isolation. The student’s ability to control their tension, anxiety, and somatic reactions is demonstrated by their personal–emotional adjustment. Institutional attachment measures how much a student identifies with the teachers and feels a connection to the organization.

Adaptation to college is a protective factor for dropping out and it is important to understand that when students enter college, they want to complete their studies, but there may be various reasons why they drop out, namely: the student transfers to another college in the same institution, chooses to go to another city to continue their studies, or wants to focus on a vocational path [16].

The concept of “satisfaction” within the academic context has been subject to various conceptualizations over the years. It has been referred to as “satisfaction with the college experience” [17], “satisfaction with the quality of instruction” [18], “satisfaction with the campus” [19], and “satisfaction with assessment” [20]. Researchers have shown interest in investigating satisfaction with specialization as it correlates with academic performance [21] and serves as a predictive factor for students’ decision to drop out of their studies [22,23,24]. The literature on self-regulation of learning behavior defines it as a collection of strategies employed by students to enhance their learning process [25,26,27,28,29,30]. Self-regulated learning has been conceptualized in various ways within the specialized literature. For instance, individuals who exhibit high levels of self-regulation set personal goals, monitor their progress during learning activities, and engage in reflective practices regarding their learning experiences [26,27,30]. Self-regulated learning encompasses three fundamental elements: regulation of learning effort, time management, and environmental management [31,32,33,34]. Extensive research has examined the interplay between satisfaction and self-regulated learning in academic contexts. Scholars have explored the extent to which satisfaction influences students’ utilization of self-regulatory strategies and how this, in turn, affects their academic outcomes. Moreover, investigations have delved into the potential reciprocal relationship, investigating how students’ self-regulatory behaviors may impact their level of satisfaction with their academic experiences.

Learning effort regulation pertains to a student’s capacity to pursue academic objectives without succumbing to fatigue and burnout. It involves maintaining an optimal level of effort that facilitates learning while avoiding excessive strain. Time management is another crucial aspect for students as they must effectively allocate their time, plan their activities, and adhere to schedules in order to succeed in their examinations. Environmental management encompasses a student’s ability to identify and establish a conducive learning environment that minimizes the influence of external distractions, such as interruptions from peers, to ensure a peaceful study atmosphere. Students who possess a high degree of self-regulated learning exhibit a range of characteristics and behaviors. They demonstrate proficiency in setting and attaining their learning goals, regularly monitor their progress, and make informed decisions regarding potential modifications to their learning strategies to enhance academic performance [34,35]. These individuals prioritize their own learning needs, engaging in actions that support their academic development while avoiding behaviors that hinder their progress [36,37].

Extensive research has explored the significance of learning effort regulation, time management, and environmental management in facilitating effective self-regulated learning. Scholars have investigated how these factors interact with students’ academic performance, motivation, and overall learning experiences. Understanding the intricate relationship between these elements is crucial for educators and institutions to provide guidance and support systems that promote optimal self-regulatory skills among students. By fostering learning environments that facilitate appropriate effort regulation, time management, and environmental conditions, educational institutions can empower students to cultivate their self-regulated learning abilities. This can be achieved through implementing strategies such as providing resources for time management and study planning, offering study spaces conducive to concentration, and promoting awareness of the importance of self-regulation in achieving academic success. Factors like learning effort regulation, time management, and environmental management are vital components of self-regulated learning. Students who effectively regulate their learning effort, manage their time, and create suitable study environments are more likely to achieve their academic goals. Understanding the significance of these factors enables educators and institutions to design interventions that foster self-regulated learning skills, empowering students to optimize their academic performance and personal growth.

Academic success involves choosing a specialization that meets students’ needs, but also their ability to have a continuity when they learn, and the in-depth study of the taught material contributes significantly to the completion of the program [1,38]. In the first year of the faculty, students have to get used to the expectations that their teachers have, the study material can be dense, and many continuous assessments occur [39,40]. Stress management must be a focus for career guidance and counseling centers within universities because a very high percentage of students have difficulty adapting to the university environment. For example, a study carried out in Australia shows us that 53% of students suffer from stress [41]. Another study shows us that the frequency of depression, anxiety, and stress among students is very high [42]. The sample consisted of 500 students, and the most common problem that students encounter is managing anxiety (88.4%). The second most prevalent is anxiety, which affects 84.4% of students, and the last place is depression, which affects 75% of students. Even though a certain level of stress is necessary to perform at an optimal level, if it is not managed properly, it can impact student performance. There are several causes of stress among students: financial problems, interaction with teaching staff, lack of support from faculty, high expectations from family, as well as teachers’ emphasis on mistakes and less on what students can improve then when learning [43,44]. There is a negative correlation between academic stress and college satisfaction [45,46]. Basically, students who cannot manage themselves will be less satisfied with their academic environment. A high level of stress also leads to decreased academic engagement.

Perceived acceptance from significant others is defined as a set of relatively stable cognitions in which the individual believes that others will unconditionally care and value them [47]. The belief that others will be with us always comes from our attachment experiences [48]. Students who receive support from family and friends will use coping strategies focused on problem solving and seek professional help in academia [49,50,51]. Parental involvement in students’ adjustment to college can have both positive and negative effects. Extremely high involvement can predispose youth to depression and anxiety [52,53]. This occurs when parents start doing activities for the youth (doing homework) to help them cope in college. In this case, the self-efficacy of young people will decrease, because they will believe that they cannot manage the situation and that the family will always make decisions on their behalf. On the other hand, if parents do not provide enough assistance and psychological support, then young students will suffer, therefore, a balance must be maintained. Stress can be more easily managed when students receive support from significant others [54].

Self-efficacy is the person’s belief that they have the necessary skills to achieve their goals and complete their tasks [55]. Academic self-efficacy is the strongest predictor of college adjustment [56,57]. In concrete terms, students who are confident in their abilities will have higher grades in college and will put more effort into understanding the information they are being taught. Self-efficacy also serves as a predictor because it influences the grades students will achieve in their first semester of college [58]. It is also a predictor for students’ intention to continue their studies [59]. Students have a number of academic demands to meet, such as exam preparation, as well as pressure to achieve high grades for scholarship [60,61] and a low level of self-efficacy that predisposes students to increased levels of anxiety, substance abuse, and insomnia [62]. College adjustment has been studied by many researchers in the educational sciences and has been measured both as a predictor and as a dependent variable. Academic adjustment is influenced by a number of demographic variables [63], prior performance [64], and students’ experiences on campus [65]. In turn, academic adjustment is a predictor of the grades that students will achieve in the session and the decision to continue their studies [66,67,68,69].

The study conducted by [70] employed an expectancy–value–cost approach to predict educational outcomes. By integrating elements of expectancy–value theory, the findings of this study extended the theoretical framework by highlighting the importance of considering three key factors: expectation, task value, and cost. These factors were identified as essential predictors of students’ academic motivation and educational success. The expectancy–value–cost approach recognizes that students’ motivation and achievement are influenced not only by their expectations of success, but also by the perceived value of the tasks they engage in and the associated costs involved. Expectancy refers to students’ beliefs regarding their ability to succeed in a particular academic endeavor. Task value encompasses the subjective significance and personal relevance students attach to their educational activities. Cost refers to the perceived negative aspects or sacrifices students may encounter in pursuing their academic goals. By incorporating all three dimensions into the predictive model, the study offered a more comprehensive understanding of the complex interplay between students’ motivation, expectations, and educational outcomes. It emphasized the need to consider not only students’ belief in their capabilities but also their subjective perceptions of task value and the potential costs associated with their academic pursuits.

Based on all the aforementioned findings, our aim is to explore the influence of variables, namely satisfaction with specialization, self-regulated learning, perceived stress among students, and perceived acceptance from family and friends, on the decision to persist in pursuing studies within the context of the Romanian higher educational system. In the present study, we commence with the hypothesis that perceived acceptance from both family and friends serves as a substantial predictor of students’ inclination to persist in their educational pursuits. Furthermore, we aim to empirically examine the potential mediating role of academic self-efficacy in the association between specialization satisfaction and academic adjustment.

This research will further test the following hypotheses:

**Hypothesis 1 (H1).**  *Students’ course completion intention is predicted by satisfaction with specialization, self-regulated learning, perceived stress, perceived acceptance from both family and friends, and academic self-efficacy.*

**Hypothesis 2 (H2).**  *Academic self-efficacy mediates the relationship between specialization satisfaction and academic adjustment.*

## 2. Materials and Methods

### 2.1. Participants

A total of 144 valid and consented responses were registered between October–December 2022 from non-randomized Romanian students majoring in Psychology, based on the convenience sampling technique. We conducted a thorough power analysis, taking into consideration several factors such as the desired confidence level, margin of error, and effect size. The selected sample size of 144 participants was determined to be appropriate based on these considerations. The margin of error was calculated based on the desired precision level of the estimates, ensuring a reliable representation of the population.

The average of age of the 144 first-year students in Psychology is 21 years old. Out of the total sample, 92.4% identified themselves as females and 7.6% identified as males. From the perspective of occupational status, 86.1% of students do not currently have a job and only 13.9% are working in different jobs. Additionally, 70.8% of students live in urban areas and 29.2% in rural areas.

All respondents gave their consent to participate in this research and agreed to aggregated data publication of their responses.

### 2.2. Measures

The Student Adaptation to College Questionnaire (SACQ) [15,71] is a 67-item questionnaire that measures the degree to which students have adjusted to college. This questionnaire has 4 subscales using a Likert rating scale from 1 (does not apply to me at all) to 9 points (applies to me perfectly). The academic adjustment subscale measures a student’s success in managing the various educational demands characteristic of the college experience: “I keep up with the learning, papers, and assignments received in classes”. Students who score high on the academic adjustment subscale put effort into academic activities and demands. Low scores are associated with a lower grade point average in the first year of study, a longer period of minimal adjustment to academic demands. Student scores are graphed in a SACQ profile on the TestCentral platform and can be downloaded as an Excel document. The academic adjustment subscale, which consists of 24 items, was employed in this study, and the Cronbach’s Alpha coefficient was 0.84.

In this study, items from the Self-Efficacy Scale [55] were adapted to refer to academia and were further referred to academic self-efficacy. The scale consists of 10 items and can be administered both individually and in groups: “Thanks to my skills, I can deal with unforeseen situations”. The scale measures the degree to which students have the ability to successfully perform certain tasks to achieve their goals. Responses are rated on a Likert scale from 1 (strongly disagree) to 4 (strongly agree). Students who score high on self-efficacy will optimally allocate the resources needed to successfully solve tasks. Students who score low on self-efficacy will avoid initiating tasks and will have the belief that they are incompetent to accomplish the task. In this study, the Cronbach Alpha coefficient is 0.845.

The College Stress Inventory [72] consists of 27 items that identify causes of stress for students (e.g., asking questions in class, writing an essay) and are rated on a Likert scale from 0 (not at all stressful) to 10 (totally stressful). This scale assesses the extent to which students experience stress when faced with college-related situations. Cronbach’s alpha in this study is 0.957.

The Motivated Strategies for Learning Questionnaire (MSLQ) [73] consists of 44 items, from which we have used only 12, representing 2 subscales: perceived effort in tasks, and time management and study environment. The first 4 items measure how students perceive effort in tasks, and the next 8 items measure time management and study environment. The answers to the items are rated on a Likert scale from 1 (does not characterize me at all) to 7 (characterizes me completely): “Even if the course materials are boring, I work on them until I complete them”. People who score high on this scale set goals for their learning even if they are not attracted to all subjects in college, they manage their study time well to complete the proposed tasks on time. In this study, we introduced the global score of self-regulation learning. Cronbach’s alpha calculated coefficient is 0.821.

Satisfaction with the specialization was measured by 2 items: “I am satisfied with the chosen specialization”. (Likert scale from 1—strong disagreement to 5—strong agreement). “Looking back, I wish I had chosen a different major (reverse coding)”. (Likert scale from 1—strongly disagree to 5—strongly agree). Cronbach’s alpha calculated coefficient is 0.872.

The evaluation of the intention to complete the studies in the Psychology specialization was measured by the following question: “Do you intend to complete the studies?” Responses were rated on a Likert scale from 1 (I am thinking of interrupting my studies) to 5 (I am determined to complete my studies). The questions regarding satisfaction with the specialization and the intention to complete the studies were inspired from the study carried out by [74].

Perceived Family and Friends Rating Scale (PAS) [75] consists of 24 items to assess the degree to which students feel accepted in their relationships with friends and family. Responses are rated on a Likert scale from 1 (strongly disagree) to 5 (strongly agree). Cronbach’s alpha for the Perceived Acceptance from Family subscale is 0.888, and for the Acceptance from Friends subscale, it is 0.750.

### 2.3. Procedures

The present study aimed to investigate the influence of various variables on the decision to persist in pursuing studies within the Romanian higher educational system. Specifically, the variables of interest included satisfaction with specialization, self-regulated learning, perceived stress among students, perceived acceptance from family and friends, and academic self-efficacy. The primary objective was to explore the potential predictive role of perceived acceptance from both family and friends in students’ inclination to persist in their educational pursuits. Additionally, the study sought to empirically examine the mediating effect of academic self-efficacy in the association between specialization satisfaction and academic adjustment.

To accomplish these objectives, a quantitative research methodology was employed. Data was collected from a sample of students enrolled in the Romanian higher educational system. Participants completed self-report measures assessing satisfaction with specialization, self-regulated learning, perceived stress, perceived acceptance from family and friends, academic self-efficacy, course completion intention, and academic adjustment.

In this research study, the sample comprised first-year students who were in the second semester of the academic year. The invitation to participate in the study was distributed during the initial week of college. Prior to administering the questionnaires, ethical approval was obtained from the Center of Research Development and Innovation in Psychology of Aurel Vlaicu University of Arad, Romania. Individual students who expressed interest in participating were provided with a link to access Google Forms, which contained pertinent information regarding the study objectives, estimated completion time for the scales, and assurance of confidentiality. The invitation explicitly stated that participants had the right to withdraw from the research at any point, and all collected data would be treated anonymously to ensure confidentiality.

The collected data were analyzed using appropriate statistical techniques. Regression analysis was utilized to examine the predictive relationship between the variables of interest (satisfaction with specialization, self-regulated learning, perceived stress, and perceived acceptance from family and friends) and students’ course completion intention. Mediation analysis, employing techniques such as bootstrapping, was conducted to assess the mediating role of academic self-efficacy in the relationship between specialization satisfaction and academic adjustment.

The study’s hypotheses were tested based on the analysis of the gathered data. Hypothesis 1 posited that students’ course completion intention would be predicted by satisfaction with specialization, self-regulated learning, perceived stress, perceived acceptance from both family and friends, and academic self-efficacy. Hypothesis 2 proposed that academic self-efficacy would mediate the relationship between specialization satisfaction and academic adjustment.

Through the utilization of this methodology, the study aimed to provide empirical evidence regarding the influences of various factors on students’ decision to persist in their studies within the Romanian higher educational system. The findings would contribute to a deeper understanding of the role of satisfaction with specialization, self-regulated learning, perceived stress, perceived acceptance, and academic self-efficacy in students’ educational experiences and outcomes.

### 2.4. Analysis Plan

Process-based psychology theory recognizes that human behavior is not just the result of static personality traits or environmental factors but is constantly influenced and shaped by ongoing processes. By focusing on processes rather than static traits or factors, process-based psychology theory offers a more dynamic and nuanced understanding of human behavior. This approach also allows for a greater understanding of how behavior can change over time, as processes interact and shift in response to new experiences and circumstances. Overall, a process-based psychology theory approach offers a comprehensive and flexible framework for understanding the complex and dynamic nature of human behavior, like in our case, understanding students’ intention to successfully complete their courses, in order to prevent early academic dropout.

The acquired data were analyzed on a MacBook using SPSS (version 28.0.1.1). For the research variables, means and standard deviations were determined. The frequencies and percentages of categorical variables were also calculated. In this study we used multiple hierarchical regression which allows us to evaluate the cumulative influence of the predictors on the dependent variable. Considering that the regression model includes several variables, it was possible to make several comparisons, which led to the minimization of type I errors. Finding a link between a collection of independent factors or predictors and a single dependent variable was the goal of the data analysis. We used t-tests to compare each independent variable to the dependent variable, and beta coefficients were used to assess each predictor’s contribution to the outcome.

## 3. Results

Table 1 shows the means and standard deviations for each variable as well as the correlation coefficients between the research variables, and Table 2 depicts detailed description of descriptive statistics for each variable, for a better understanding of results ranges.

Participants demonstrated a high level of academic adjustment, with a mean score of 95.3. Academic adjustment encompasses an individual’s ability to adapt to the academic environment, effectively manage their time, and cope with the demands of their studies. The high score suggests that participants exhibited strong skills in these areas, indicating a successful adaptation to the academic context. Participants reported a moderate level of academic self-efficacy, as indicated by a mean score of 32.8. Academic self-efficacy reflects an individual’s belief in their capability to perform academic tasks and achieve desired outcomes. The moderate score suggests that participants possessed a moderate level of confidence in their academic abilities. The mean score of 9.19 indicates a relatively high level of satisfaction with studies among participants. This finding suggests that individuals in the sample generally experienced contentment and fulfillment in their academic pursuits. Factors contributing to this satisfaction may include engaging course content, effective teaching quality, and a positive learning environment. Participants demonstrated a moderate level of intention to persist in their academic pursuits, as reflected by a mean score of 4.82. This result suggests that individuals in the sample possessed a moderate level of commitment and motivation to continue their studies despite potential challenges or obstacles. Participants exhibited a moderate level of self-regulation in study behavior, with a mean score of 60.8. Self-regulation pertains to an individual’s ability to plan, organize, and regulate their study habits and academic activities. The moderate score suggests that participants displayed a moderate level of effectiveness in managing their study behaviors. Participants reported a relatively high level of perceived stress, as indicated by a mean score of 139. Perceived stress captures individuals’ subjective perception of the level of stress they experience in their academic lives. The higher score suggests that participants perceived their academic environment as quite stressful, possibly due to academic demands, time constraints, or other related factors. Participants reported a moderate level of perceived acceptance from family members, with a mean score of 44.3. Perceived acceptance from family refers to individuals’ perception of the level of acceptance and support they receive from their family regarding their academic endeavors. The moderate score suggests that participants felt moderately accepted and supported by their family members in relation to their academic pursuits. Participants reported a relatively high level of perceived acceptance from friends, as indicated by a mean score of 84.7. Perceived acceptance from friends reflects individuals’ perception of the level of acceptance and support they receive from their friends in relation to their academic pursuits. The higher score suggests that participants perceived a high level of acceptance and support from their friends. In terms of correlations, academic self-efficacy demonstrated a significant positive correlation with satisfaction with studies (r = 0.282, *p* < 0.001), indicating that participants with higher levels of academic self-efficacy tended to experience greater satisfaction with their studies. However, it did not show significant correlations with other variables. Intention to persist displayed a significant positive correlation with academic self-efficacy (r = 0.355, *p* < 0.001), suggesting that individuals with higher levels of academic self-efficacy were more likely to exhibit a stronger intention to persist in their academic pursuits. Self-regulation study behavior exhibited significant positive correlations with academic self-efficacy (r = 0.337, *p* < 0.001), satisfaction with studies (r = 0.330, *p* < 0.001), and intention to persist (r = 0.282, *p* < 0.001). These findings indicate that participants who demonstrated higher levels of self-regulation in their study behavior tended to report higher levels of academic self-efficacy, satisfaction with studies, and intention to persist. Perceived stress displayed a significant negative correlation with self-regulation study behavior (r = −0.191, *p* < 0.05), suggesting that individuals who perceived higher levels of stress tended to exhibit lower levels of self-regulation in their study behavior. Perceived acceptance from family demonstrated significant positive correlations with academic self-efficacy (r = 0.477, *p* < 0.001), satisfaction with studies (r = 0.477, *p* < 0.001), and intention to persist (r = 0.179, *p* < 0.05). These results suggest that participants who perceived higher levels of acceptance from their family members tended to report higher levels of academic self-efficacy, satisfaction with studies, and intention to persist. Perceived acceptance from friends exhibited significant positive correlations with academic self-efficacy (r = 0.525, *p* < 0.001), satisfaction with studies (r = 0.525, *p* < 0.001), and intention to persist (r = 0.217, *p* < 0.01). This implies that participants who perceived higher levels of acceptance from their friends tended to report higher levels of academic self-efficacy, satisfaction with studies, and intention to persist.

In summary, the results indicate that participants in this study demonstrated a high level of academic adjustment and satisfaction with their studies. They also displayed moderate levels of academic self-efficacy, intention to persist, and self-regulation in study behavior. However, participants reported a relatively high level of perceived stress. Moreover, they perceived moderate acceptance from family members and high acceptance from friends in relation to their academic pursuits. These findings provide valuable insights into the various factors influencing academic adjustment and psychological well-being among individuals in an academic context.

To test the first hypothesis, hierarchical multiple regression analysis was used. Table 1 shows correlation coefficients, whereas Table 3 shows regression coefficients. Intention to complete studies is predicted by satisfaction with the specialization (R^2^ = 0.113). In the second step, we added self-regulation learning and students’ perceived stress, and the explained variance increased (R^2^ = 0.187). In the third step, we added the variables perceived acceptance from family and perceived acceptance from friends, and the variance of the dependent variable reached 0.189. In the fourth step, we added academic self-efficacy and the variance of the dependent variable reached 0.195.

In step 1, satisfaction with specialization explained 11.3% (R² = 0.113) of the variance in intention to complete studies. Adding self-regulated learning and students’ perceived stress in step two, explains an additional 7.4% of variance (R² = 0.074) and makes the prediction model consisting of satisfaction with the specialization, self-regulated learning, and perceived stress by students to be predictive of intention to complete studies. Adding family acceptance and friends’ acceptance in step 3 explained an additional 0.2% of the variance, while not being significant predictors of intention to complete studies. Adding academic self-efficacy in step 4 explained an additional 0.6% of the variance, while not being a significant predictor of intention to complete studies.

Based on regression Model 2 we can confidently estimate the level of intention to complete studies F (3, 140) = 10.714, *p* < 0.001, providing significantly better predictions than those we could make based on results from Model 1. Based on regression Model 3, we can confidently estimate the level of Intention to complete studies F (5, 138) = 6.432; *p* < 0.001, and based on the regression Model 4, we can confidently estimate the level of Intention to complete studies F (6, 137) = 5.535; *p* < 0.001.

All six predictors: satisfaction with specialization, self-regulated learning, student perceived stress, family acceptance and friends’ acceptance, and academic self-efficacy in a single model explain 19.5% (R² = 0.195, *p* = 0.006) of the variance of students’ intention to continue their studies, representing an important proportion of explained variance in our dependent variable.

**Figure 1:**
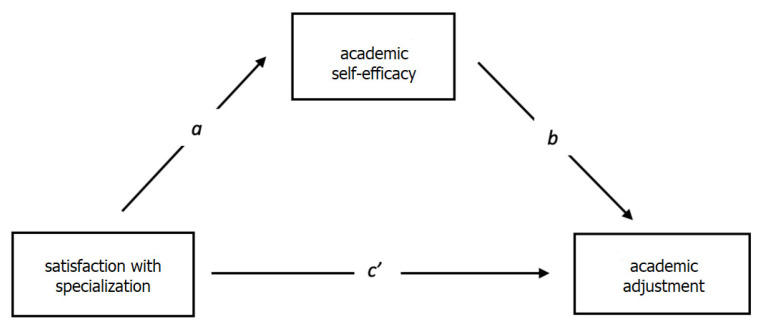
A model of mediation for the investigated variables: academic self-efficacy, satisfaction with specialization, and academic adjustment.

The results in Table 4 and Table 5 and Figure 1 show that academic self-efficacy partially mediates the relationship between specialization satisfaction and academic adjustment (R = 0.3138, R^2^ = 0.09, F = 15.5131, *p* = 0.001).

It appears that there is a small R-squared (R^2^) value in the mediation analysis. The R-squared value represents the proportion of variance in the dependent variable that can be explained by the independent variable and the mediator variable. In this case, the R-squared value is R^2^ = 0.09, indicating that approximately 9% of the variance in academic adjustment can be explained by the combined influence of specialization satisfaction and academic self-efficacy. The small R-squared value suggests that there may be other factors or variables not included in the analysis that contribute to academic adjustment. It is important to note that the R-squared value can be influenced by various factors, including the complexity of the relationship being studied, the number and strength of the predictors, and the variability of the data. In mediation analyses, the focus is typically on the indirect effects and the significance of the mediation pathway, rather than the overall amount of variance explained.

The total effect of the independent variable on the dependent variable is 2.7840. However, the *p*-value is greater than 0.05, indicating that the total effect is not statistically significant. The direct effect of the independent variable on the dependent variable is 1.6443. Similarly, the *p*-value is greater than 0.05, suggesting that the direct effect is not statistically significant. The relationship between the independent variable and the dependent variable is not significant, as both the total and direct effects are non-significant. The indirect effect of the independent variable on the dependent variable through the mediator variable is 1.1397. The confidence intervals (lower bound: 0.0201, upper bound: 2.6449) indicate that the indirect effect is statistically significant as it does not include zero. Based on these results, the conclusion is that there is partial mediation.

The independent variable, satisfaction with specialization, does not have a significant direct effect on the dependent variable, academic adjustment, but it influences the dependent variable indirectly through the mediator variable, academic self-efficacy. Therefore, the mediation process is partial, as the indirect effect is significant while the direct effect is not [76]. The main conclusion to our mediation analysis is satisfaction with specialization does not represent a significant predictor to students’ academic adjustment, only academic self-efficacy.

## 4. Discussion

The study procedures involved selecting first-year students from the second semester and inviting them to participate in the study during the first week of college. Prior to distributing the questionnaires, the Ethics Committee of the Center of Research Development and Innovation in Psychology of Aurel Vlaicu University of Arad, Romania provided consent for the study. The students were informed about the study’s objectives, the time required to complete the scales, the confidentiality of their responses, and their right to withdraw from the research at any time. The collected data were analyzed using SPSS version 28.0.1.1, and descriptive statistics and correlations between the research variables were examined. To test the first hypothesis, a hierarchical multiple regression analysis was conducted. The results of the regression analysis indicated that satisfaction with specialization explained 11.3% of the variance in the intention to complete studies in the first step. In the second step, the inclusion of self-regulated learning and students’ perceived stress accounted for an additional 7.4% of variance. In the third step, the addition of family acceptance and friends’ acceptance explained an additional 0.2% of variance, which was not a significant predictor of the intention to complete studies. Adding academic self-efficacy in step 4 explained an additional 0.6% of the variance, while not being significant predictor of intention to complete studies.

Ultimately, when all six predictors were included in a single model, they accounted for 19.5% of the variance in students’ intention to continue their studies. The analysis plan of the study included t-tests to compare each independent variable with the dependent variable and beta coefficients to assess the contribution of each predictor to the outcome. The findings revealed that satisfaction with specialization, self-regulated learning, student perceived stress, family acceptance and friends’ acceptance, and academic self-efficacy collectively explained a substantial proportion of the variance in the dependent variable.

The primary objective of this research was to identify key factors that influence student retention in order to develop intervention techniques for at-risk students in the psychology department and the career counseling and assistance center. Consistent with our results, previous studies [77,78,79] have found that academic adjustment mediates the relationship between satisfaction with specialization and the intention to continue studies. Based on the premise that students who are satisfied with their choice of studying Psychology are more likely to complete their studies, as their expectations about the faculty have been met, we proposed a mediating relationship.

Specifically, we hypothesized that academic self-efficacy mediates the relationship between satisfaction with specialization and academic adjustment. Results show that the independent variable satisfaction with specialization does not have a significant direct effect on the dependent variable academic adjustment, but it influences the dependent variable indirectly through the mediator variable academic self-efficacy. Therefore, the mediation process is partial, as the indirect effect is significant while the direct effect is not [76]. The main conclusion to our mediation analysis is satisfaction with specialization does not represent a significant predictor to students’ academic adjustment, only academic self-efficacy.

In a related study, [80] investigated the mediating role of academic adjustment in the relationship between students’ perceptions of social support and life satisfaction. The regression analysis results indicated that academic adjustment significantly mediated the relationship between perceived social support and life satisfaction. Social support systems from family and teachers can enhance the academic adjustment and life satisfaction of first-year students. Moreover, according to the findings of [81], academic success and college connectedness were predictors of life satisfaction, whereas academic self-efficacy and college appreciation were not. Conscientiousness was found to predict academic self-efficacy, and college well-being predicted self-reported achievement, while anxiety and depression did not. This study emphasizes the importance of understanding the individual factors that influence success and happiness during students’ transition to university.

This research makes a significant contribution as one of the pioneering studies conducted in Romania to identify significant predictors that influence students’ decision to persist in their educational pursuits. A unique aspect of our study is the inclusion of the stress variable in our predictive model to examine its contribution to explaining the variance in students’ successful completion of their studies. We recognize the relevance and impact of stress on students’ academic journey, particularly during the critical transitional phase of the first year of college. This period is known to be associated with increased stress levels, as evidenced by the high number of students seeking assistance at the university’s career guidance and counseling center [82,83,84,85]. By incorporating the stress variable, we aimed to investigate its extent of explaining variance in students’ ability to successfully complete their studies. It is worth noting that many students face challenges in effectively managing their time and organizing their learning materials, which often manifests as difficulties in adapting to the demands of higher education. This observation underscores the significance of our research, as it addresses a crucial gap in understanding the factors that influence students’ persistence and academic success.

By shedding light on the predictors of students’ decision to continue their studies, including the influence of stress and the challenges associated with time management and learning organization, our research provides valuable insights for both academia and student support services. This knowledge can inform the development of targeted interventions and support systems aimed at equipping students with effective stress management strategies, time management skills, and organizational techniques to facilitate a smoother transition into and progression through their academic journey.

The results show us that when students are satisfied with their specialization choice, psychology in our case, they will have more confidence in their academic abilities, and this will help them to be efficient and put effort into their assignments. Students who enter college and perceive that their specialization is consistent with their professional interests will be more satisfied with their choice of degree program and will attend classes and seminars [86]. Additionally, [87] highlighted the fact that students in their individual study process do not only acquire theoretical information and skills, but also develop personally. We know that the learning environment and time efficiency have a key role in the self-regulated learning, and this must be a starting point for faculty psychologists if they want to help students achieve better academic performance.

In [88], the factors that are related to student satisfaction were examined and indicators such as support provided by teachers and perceived acceptance from the family were identified, but “physical” factors (study materials, dormitories, number of rooms) also matter a lot. Academic self-efficacy is related to efforts and persistence in the learning process with self-regulated learning [89]. Adapting to the first year of studies is a longer process, and students have to make a number of changes in their lives. The first major change for many students is that they have to move to another city, and that means becoming independent. The results obtained show us that when students are satisfied with the study program, they will have confidence in their abilities and adapt more easily to the academic environment.

The results of this study are consistent with the study carried out by [90] who identified associations between academic self-efficacy, major satisfaction, and academic adjustment. Last but not least, in this study we wanted to investigate the role of motivational and behavioral factors in explaining the intention to complete studies. The obtained results highlight some interesting aspects. Students who are satisfied with their choice of Psychology major will be more motivated to complete their studies. All definitions of academic success have a multidimensional nature and include cognitive aspects (skills, abilities of the student), behaviors that are reflected in acquired skills and the emotional dimension that includes satisfaction with the specialization that is a decisive factor in continuing studies. In other words, individuals feel satisfied with a domain when they feel competent in tasks and anticipate favorable outcomes [91,92]. The ability to self-regulated learning is another significant predictor of intention to complete studies. Even though cognitive skills play an important role in predicting academic performance and the intention to complete studies, non-cognitive factors have been identified that influence students to reach the third year, including self-regulated learning. If, from the first year, the students make a study schedule, benefit from an optimal learning environment where they are not distracted by external sources (phone, social networks), and make efforts to understand the theory, then they have a good chance of reaching the year three and receiving their license. Perceived academic stress influences the intention to complete college, the relationship being a negative one. The higher the perceived stress, the more students are at risk of dropping out. In the literature, there are many studies that show us that stress in students is related to depression and anxiety [93,94]. There are differences between men and women when it comes to academic stress, with men being more reluctant to talk about their inner states [95]. Educational psychologists working in counseling centers at higher education institutions must be prepared to assess the level of academic stress perceived by students when they want to carry out counseling interventions. For example, counselors and professionals can offer academic workshops and seminars that explain to students in detail the emotional changes they will go through when entering college. Students can also obtain a sense of what is expected of them academically by working with student support services and faculty members [96].

Another study conducted by [97] shows us that students who participate in support and career development courses will have a higher level of academic adjustment and self-efficacy. Another result of this study shows that students who were initially undecided and did not know what to do with their future had a clearer picture of their future profession after participating in these courses. Students need socialization, and research in recent years has emphasized learning communities in increasing students’ academic adjustment [98,99]. Learning community refers to organized groups of students who support each other in academic tasks and other issues related to student life. They have been successful in helping to develop academic and interpersonal skills, such as communication and problem-solving skills, and have been shown to be effective in improving institutional commitment, performance, and academic success [100,101].

Career guidance courses and seminars can be designed by specialists to convey information to students about their field, the challenges they will face, and older students can help first-year students better integrate the information into learning communities. Combining career development courses and learning communities can be more effective if done together and have positive educational outcomes. Communication between the teacher and students is another variable of interest in the prevention of university dropout. A study [102] showed that strategies to improve academic adjustment led to record results: 90.4% of first-year students made it to the second year. The measures that contributed to this success are given by the efforts of the teaching staff to communicate frequently with the first-year students and to show their willingness to offer them a helping hand when they had uncertainties.

Stress was significantly connected with institutional support for first-generation students but not for continuing-generation students [103]. The relationship between stress and perceived academic goal attainment for first-generation college students was also clarified by institutional assistance, but not for continuing students. Reflective coping was used to explain the connection between stress and first- and second-generation college students’ perceptions of their ability to achieve academic goals. Contrary to expectations, the presence of friends and family did not explain the relationship between stress and perceived academic goal attainment for first- or continuing-generation college students. According to the findings, institutional supports are somewhat important for understanding how stress and first-generation college students’ evaluations of their academic achievement are related [103].

### 4.1. Limitations

It is also important to acknowledge the limitations of this study. Firstly, the cross-sectional nature of our investigation limits our ability to establish causality between the variables examined. Longitudinal studies would provide a more in-depth understanding of the dynamic nature of academic persistence and allow for the identification of critical periods for targeted interventions. Additionally, the use of convenience sampling may introduce potential selection bias, limiting the generalizability of our findings. Future studies should aim for larger and more diverse samples to enhance the external validity of the results. The sample size was relatively small and limited to first-year university students in a specific context. Therefore, caution should be taken when generalizing the findings to other contexts. Additionally, the study relied on self-report measures, which may be subject to social desirability bias. Future research could use a larger and more diverse sample and incorporate objective measures of academic persistence.

### 4.2. Theoretical Implications

In terms of theoretical implications, our findings contribute to the existing body of knowledge on student persistence in higher education. By examining the relationships between satisfaction with specialization, self-regulated learning, perceived stress, perceived acceptance from family and friends, and the intention to complete studies, we shed light on the psychological factors that influence students’ academic persistence. Our results align with process-based psychology theories that emphasize the individual’s conscious decision-making process rather than traditional sociological models of student persistence. This highlights the need for a multidimensional approach that incorporates psychological variables in understanding student dropout phenomena.

### 4.3. Practical Implications

The findings of this research have important practical implications for student retention and intervention strategies in the psychology department and the career counseling and assistance center. Previous studies have also highlighted the role of academic adjustment in mediating the relationship between satisfaction with specialization and the intention to continue studies.

Based on the premise that students who are satisfied with their choice of studying Psychology are more likely to complete their studies, this research proposed a mediating relationship involving academic self-efficacy. The results indicate that satisfaction with specialization does not directly influence academic adjustment, but it does have an indirect effect through the mediator variable of academic self-efficacy. This suggests that academic self-efficacy plays a crucial role in explaining the relationship between satisfaction with specialization and academic adjustment.

The practical implications of these findings are two-fold. First, it is important for educational institutions and career counseling centers to recognize the significance of academic self-efficacy in students’ academic adjustment. By focusing on enhancing students’ belief in their own abilities to succeed academically, educational institutions can provide targeted interventions and support programs to promote academic persistence and success.

Second, while satisfaction with specialization alone may not directly predict students’ academic adjustment, it remains important for educational institutions to create an environment that fosters student satisfaction and engagement with their chosen field of study. This can be achieved through improving the quality of teaching, curriculum design, and providing resources and support services tailored to students’ needs and interests.

Overall, the practical implication is that academic self-efficacy should be prioritized in interventions and support programs, while also considering the importance of fostering student satisfaction with their chosen specialization. By addressing these factors, educational institutions and career counseling centers can better support students’ academic adjustment, persistence, and overall success in their academic pursuits.

## 5. Conclusions

The persistence of students in their educational journey is crucial not only for individual achievement but also for the overall effectiveness and success of educational institutions. When students prematurely discontinue their studies, it can lead to negative consequences such as lower academic attainment, decreased workforce productivity, and limited socioeconomic opportunities. Consequently, understanding the factors influencing student retention has become a prominent area of scientific inquiry.

The present study investigated the predictors of academic persistence among first-year university students, with a focus on the relationships between satisfaction with specialization, self-regulated learning, perceived stress, perceived acceptance from family and friends, and the intention to complete studies. Additionally, the partial mediating role of academic self-efficacy in the relationship between satisfaction with specialization and academic adjustment was examined. The findings provide valuable insights into understanding student dropout in the context of higher education in Romania.

Moreover, the study revealed that self-regulated learning plays a significant role in students’ intention to persist in their studies. Students who possess effective self-regulation skills, such as managing their time and maintaining focus on academic tasks, are more likely to stay engaged and motivated in their learning process. This finding underscores the importance of promoting self-regulated learning strategies among students to enhance their academic persistence.

Perceived stress was identified as another influential factor in students’ intention to complete their studies. Higher levels of perceived stress were associated with a decreased intention to persist academically. This highlights the need for universities to provide adequate support systems and resources to help students effectively manage and cope with stress during their academic journey. Interventions aimed at stress reduction and promoting mental well-being among students may contribute to higher levels of academic persistence. Furthermore, the study examined the mediating role of academic self-efficacy in the relationship between satisfaction with specialization and academic adjustment. The results demonstrated that academic self-efficacy fully mediates this relationship, indicating that students’ belief in their own abilities to succeed academically is crucial for their adjustment and persistence. Strengthening students’ academic self-efficacy through targeted interventions and support programs may foster their overall academic adjustment and increase their likelihood of persisting in their studies.

The findings of this study have important implications for both future research and practical applications. Future research endeavors should further explore the complex factors influencing student persistence, including additional psychological, social, and environmental variables. Longitudinal studies could provide insights into the developmental trajectories of academic persistence and identify critical periods for targeted interventions.

Overall, this study contributes to the existing literature on academic persistence by highlighting the significance of satisfaction with specialization, self-regulated learning, perceived stress, and academic self-efficacy. By recognizing these predictors, educators, administrators, and policymakers can develop evidence-based strategies and interventions to foster students’ academic persistence, enhance learning outcomes, and ultimately reduce student dropout rates in higher education institutions.

## Figures and Tables

**Table 1:** Descriptive statistics and correlations between research variables.

Variables	m	SD	1	2	3	4	5	6	7	8
1. Academic adjustment	95.3	27.1								
2. Academic self-efficacy	32.8	4.65	0.185 *							
3. Satisfaction with specialization	9.19	1.34	0.048	0.282 ***						
4. Intention to persist	4.82	0.575	0.027	0.104	0.355 ***					
5. Self-regulation study behavior	60.8	11.1	−0.028	0.337 ***	0.330 ***	0.282 ***				
6. Perceived stress	139	57.1	0.141	0.042	0.049	−0.191 *	0.155			
7. Perceived acceptance from family	44.3	11.1	0.014	0.477 ***	0.477 ***	0.179 *	0.380 ***	−0.039		
8. Perceived acceptance from friends	84.7	14.3	0.038	0.525 ***	0.525 ***	0.217 **	0.398 ***	−0.040	0.944 ***	

*** At the 0.001 level, there is a correlation (two-tailed). ** At the 0.01 level, there is a correlation (two-tailed). * At the 0.05 level, there is a correlation (two-tailed).

**Table 2:** Detailed descriptive statistics.

Descriptive Statistics							
	N	Range	Minimum	Maximum	Mean	Std. Deviation	Variance
1. Academic adjustment	144	89.00	37.00	126.00	95.3125	27.06286	732.398
2. Academic self-efficacy	144	19	21	40	32.76	4.655	21.668
3. Satisfaction with specialization	144	6	4	10	9.19	1.339	1.794
4. Intention to persist	144	4	1	5	4.82	0.575	0.331
5. Self-regulation study behavior	144	58	26	84	60.83	11.078	122.727
6. Perceived stress	144	250	1	251	139.13	57.121	3262.801
7. Perceived acceptance from family	144	48	12	60	44.31	11.114	123.514
8. Perceived acceptance from friends	144	76	35	111	84.74	14.322	205.119
Valid N (listwise)	144						

**Table 3:** Hierarchical Regression Predicting Intention to Persist Summary Table.

Variable	B	SE	β	*p*	R²	ΔR²
Model 1					0.113	0.113
Constant	3491 ***	0.315		<0.001		
Satisfaction with specialization	0.144 ***	0.034	0.336	<0.001		
Model 2					0.187	0.074
Constant	3341 ***	0.353		<0.001		
Satisfaction with specialization	0.113 ***	0.034	0.263	0.001		
Self-regulated learning	0.012 **	0.004	0.232	0.005		
Stress perceived by students	−0.002 **	0.001	−0.208	0.008		
Model 3					0.189	0.002
Constant	3197 ***	0.442		<0.001		
Satisfaction with specialization	0.111 **	0.035	0.259	0.002		
Self-regulated learning	0.011 *	0.005	0.213	0.016		
Stress perceived by students	−0.002 *	0.001	−0.202	0.012		
Acceptance from the family	−0.002	0.012	−0.043	0.848		
Acceptance from friends	0.004	0.009	0.090	0.693		
Model 4					0.195	0.006
Constant	3298	0.453		<0.001		
Satisfaction with specialization	0.012 **	0.036	0.279	0.001		
Self-regulated learning	0.012 *	0.005	0.224	0.013		
Stress perceived by students	−0.002 *	0.001	−0.197	0.014		
Acceptance from the family	−0.004	0.012	−0.071	0.755		
Acceptance from friends	0.006	0.010	0.1610	0.501		
Academic self-efficacy	−0.012	0.012	−0.097	0.310		

Note. *** *p* < 0.001; ** *p* < 0.01; * *p* < 0.05.

**Table 4:** A model of mediation for the investigated variables: academic self-efficacy, satisfaction with specialization, and academic adjustment.

Path	r^2^	F	df	*p*	B	SE(B)	β	*p*	95% CI
c	0.0190	2.7482	(1142)	0.0996	2.7840	1.6794	1.6578	0.0996	−0.5358–6.1038
a	0.0985	15.5131	(1142)	0.0001	1.0906	0.2769	3.9387	0.0001	0.5432–1.6380
b and c′	0.0481	3.5632	(2141)	0.309					
c					1.6443	1.7484	0.9405	0.3486	−1.8122–5.1009
b					1.0450	0.5031	2.0770	0.0396	0.0504–2.0396
axb					1.1397				

r^2^ = explained variation/total variation; F = ANOVA; B = unstandardized coefficients; (SE) = standard error; β = standardized coefficients; (df) = degree of freedom; *p* = level of significance; 95% confidence interval (CI) = 95.0% confidence interval for B.

**Table 5:** Sequential mediation summary.

Total Effect	Direct Effect	Relationship	Indirect Effect	Confidence Intervals		t = Indirect Effect /SE	Conclusion
				Lower Bound	Upper Bound		
2.7840 *p* > 0.05	1.6443 *p* > 0.05	H1: specialization satisfaction → academic self-efficacy→ academic adjustment	1.1397 *p* < 0.05	0.0201	2.6449	1.7005	Partial mediation

## Data Availability

Data will be made available on request by the first author and the corresponding author.
